# Modeling construction and demolition waste quantities in Tanta City, Egypt: a synergistic approach of remote sensing, geographic information system, and hybrid fuzzy neural networks

**DOI:** 10.1007/s11356-023-29735-8

**Published:** 2023-09-20

**Authors:** Nehal Elshaboury, Wael M. AlMetwaly

**Affiliations:** 1 Construction and Project Management Research Institute, Housing and Building National Research Centre, Giza, Egypt; 2 Department of Geography and GIS, Faculty of African Postgraduate Studies, Cairo University, Giza, Egypt; 3 GIS Expert at General Organization of Physical Planning, Ministry of Housing, Utilities, and Urban Communities, Cairo, Egypt

**Keywords:** Waste quantification, Socioeconomic analysis, Machine learning, Adaptive quantum particle swarm optimization algorithm, Hierarchical pruning, Prediction performance

## Abstract

A waste management strategy needs accurate data on the generation rates of construction and demolition waste (CDW). The objective of this study is to provide a robust methodology for predicting CDW generation in Tanta City, one of the largest and most civilized cities in Egypt, based on socioeconomic and waste generation statistics from 1965 to 2021. The main contribution of this research involves the fusion of remote sensing and geographic information systems to construct a geographical database, which is employed using machine learning for modeling and predicting the quantities of generated waste. The land use/land cover map is determined by integrating topographic maps and remotely sensed data to extract the built-up, vacant, and agricultural areas. The application of a self-organizing fuzzy neural network (SOFNN) based on an adaptive quantum particle swarm optimization algorithm and a hierarchical pruning scheme is introduced to predict the waste quantities. The performance of the proposed models is compared against that of the FNN with error backpropagation and the group method of data handling using five evaluation measures. The results of the proposed models are satisfactory, with mean absolute percentage error (MAPE), normalized root mean square error (NRMSE), determination coefficient, Kling–Gupta efficiency, and index of agreement ranging between 0.70 and 1.56%, 0.01 and 0.03, 0.99 and 1.00, 0.99, and 1.00. Compared to other models, the proposed models reduce the MAPE and NRMSE by more than 92.90% and 90.64% based on fivefold cross-validation. The research findings are beneficial for utilizing limited data in developing effective strategies for quantifying waste generation. The simulation outcomes can be applied to monitor the urban metabolism, measure carbon emissions from the generated waste, develop waste management facilities, and build a circular economy in the study area.

## Introduction

The building industry is one of the crucial sectors of economic development in every country (Kittinaraporn et al. 2022). A third of all energy generated is consumed by the building industry, which also produces 40% of carbon dioxide emissions to the environment (Luangcharoenrat et al. 2019). Moreover, the building sector consumes about 3 billion tons of the world’s raw materials annually (Guerra and Leite 2021). The waste produced by construction, renovation, or demolition operations, referred to as construction and demolition waste (CDW), represents 30% of the overall solid waste produced globally (Purchase et al. 2022; Ding et al. 2023). In Egypt, the generated CDW quantities are estimated to be 50 million tons annually, increasing by 5 million tons per year (Albawabh News 2023). This waste stream comprises inert and noninert components (e.g., glass, steel, wood, and concrete), and it occupies landfill space, causes considerable environmental degradation, generates geologic risks, and has other negative impacts (Jain et al. 2020; Kabirifar et al. 2020; Elshaboury and Marzouk 2021). Hence, governments and municipal authorities must manage CDW effectively; however, the main obstacle that hinders waste reuse and recycling in Egypt is the absence of accurate estimates of their quantities. Recycling offers numerous benefits for sustainable development across societal, environmental, and economic dimensions (Wei et al. 2023). Many waste management approaches, such as planning landfill space, adopting disposal levies or recycler subsidies, and establishing waste management plans, require accurate estimates of waste generation rates (Yu et al. 2021; Hua et al. 2023).

The assessment of waste generation at both the project and regional levels has attracted attention on a global scale. Elshaboury et al. (2022) conducted a bibliometric and scientometric study on 895 publications related to CDW management. The study found that management strategies, as well as estimation and quantification, are the primary emerging themes in this field. Estimation is essential in developing countries where no formal recording systems regularly gather and publish data on waste generation rates (Lu et al. 2021). According to Gao et al. (2018), basic and comprehensive methods can be applied to predict CDW generation, including site visits (SV), generation rate calculations (GRC), variable modeling (VM), classification system accumulation (CSA), material flow analysis (MFA), geographic information system (GIS), and building information modeling (BIM). Each approach for estimating CDW generation has advantages and disadvantages. The SV technique necessitates performing field surveys, including direct measurement by weighing or measuring volume, as well as indirect measurement by utilizing other indicators, such as haulage tickets (Hoang et al. 2020). Using the GRC technique, the total waste volume may be estimated by multiplying the generation rate of a given unit by the associated quantity (Hu et al. 2023). VM can model the relationships between input variables and targeted output using machine learning, system dynamics, and other modeling approaches. The CSA approach quantifies each material by combining the GRC method with a waste categorization (Hu et al. 2021). MFA may assess the input and output of building materials and determine the flow of materials during the entire construction activity (Abdelshafy and Walther 2022). Other approaches, such as employing GIS and BIM, do not fall into any of the abovementioned categories. This study will employ GIS and VM techniques for reliable prediction of regional waste generation.

The major objective of this research is to predict the CDW quantities in Tanta City, which is the second most populous city in the world, the third-most civilized Egyptian city, and the largest city in the Delta (Egypt Independent 2022). The spatial data are acquired from the city maps and classification of satellite images after validation to extract the utilized factors, which are used as inputs for the model development. The proposed models incorporate the application of a self-organizing fuzzy neural network (SOFNN) modeling approach based on an adaptive quantum particle swarm optimization (AQPSO) algorithm and a hierarchical pruning scheme (HPS). The performance of the AQPSO-SOFNN and SOFNN-HPS models is evaluated by considering socioeconomic, urban growth, and waste-generation statistics. The novelty of this research is the integration of remote sensing and geographic information systems to establish a geographical database, which is employed for predicting the generated waste quantities using machine learning. Moreover, it is the first application of AQPSO-SOFNN and SOFNN-HPS for modeling and quantifying CDW volumes. Additionally, the performance of these models is compared with other state-of-the-art competitors, including FNN with an error backpropagation (FNN-EBP) learning approach and group method of data handling (GMDH). As for the verification and evaluation of prediction models, K-fold cross-validation is conducted to verify the proposed models. Moreover, performance measures that reflect the relationship between observed and predicted values are used to assess the performance of predictive models. The research objective is aligned with Egypt Vision 2030, which targets a substantial improvement in the collection and efficiency of solid waste (MPED 2023). It also serves waste management law No. 202 of 2020, which emphasizes the importance of developing a database that records the quantities of various types of waste and the existing and new landfills in each governorate (Official Gazette 2022). Furthermore, providing a robust methodology for quantifying the waste volumes is crucial to the operation of 38 crushers that were allocated at a total cost of 518.6 million pounds. These crushers aim to facilitate the reuse of CDW in all Egyptian governorates to develop an effective and sustainable waste management system.

## Quantifying CDW generation

Estimating CDW generation has been acknowledged as a cornerstone for waste management. In recent years, machine learning models have been widely employed to predict CDW generation. These models include support vector regression (SVR), artificial neural network (ANN), random forest (RF), K-nearest neighbor (KNN), multiple linear regression (MLR), support vector machine (SVM), and decision tree (DT). Song et al. (2017) developed a gray model-SVR model that enhanced the gray model’s forecasting performance by modifying the residual series using the SVR technique and a transition matrix for predicting the CDW in China. The application of the proposed model allowed for examining the volume, components, and distribution of waste in various Chinese provinces. Akanbi et al. (2020) developed deep neural network models to forecast the salvage and waste material from a demolished building. Datasets for the model development were gathered from 2280 building demolition data in the UK. The models were developed to forecast the quantity of reused, recycled, and landfilled materials. The building characteristics, including the number of floors, building volume, gross floor area, building type (i.e., office, retail, and education), and building type (i.e., steel, concrete, masonry, and timber), were among the input variables. The mean determination coefficient (*R*^2^) was 0.97, while mean absolute error (MAE) values ranged from 17.93 to 19.04. Four building design scenarios were used to test the models. The developed models exhibited high accuracy in estimating the salvage materials given the building’s basic characteristics.

Cai et al. (2020) proposed a hybrid intelligent strategy that combined SVR, single spectrum analysis (SSA), and long short-term memory network (LSTM) networks to predict CDW generation rates in Hong Kong. The trend and fluctuation in time series data were captured using SSA, and the SVR and LSTM models were then employed and aggregated for better prediction results. The optimal model parameters were defined using grid search optimization. The results revealed the outperformance of the proposed approach against the benchmark methods and its viability for CDW generation forecasting. Huang et al. (2020) developed a time-series forecasting approach based on the LSTM network to anticipate construction waste in Shanghai and Hong Kong. The proposed LSTM model exhibited better performance when compared to existing time-series forecasting models, including SVR, ridge regression, and ANN. Moreover, the overfitting problem of the proposed model was addressed by adding a dropout layer to enhance its generalization performance. The findings demonstrated the model’s superior ability to address univariate nonlinear forecasting problems. Lu et al. (2021) quantified the construction waste generation in Greater Bay, China, using the data acquired by the local government agencies. These data included building-related, socioeconomic, and CDW generation statistics from 2005 to 2019. Four different machine learning models were used to analyze the data: MLR, ANN, DT, and Grey model. The developed models yielded *R*^2^ values ranging from 0.756 to 0.977 throughout the testing phase. According to this analysis, the Greater Bay generated around 364 million m^3^ of building waste in 2018.

Cha et al. (2020) employed RF to estimate demolition generation based on small datasets. The model accounted for building structure (e.g., masonry, wood, and reinforced concrete), building use (e.g., residential, commercial, and residential/commercial), region, wall material (e.g., reinforced concrete, brick, block, and soil), roofing material (e.g., slab, slab and roofing tile, roofing tile, and roof with asbestos), and gross floor area. The results showed the capability of the RF prediction model (Pearson’s correlation coefficient: *R* = 0.69–0.87 and *R*^2^ = 0.55–0.80) to deal with a small dataset of demolition waste. In another study, Cha et al. (2022a) incorporated categorical principal components analysis (CATPCA) with the SVR and ANN techniques to improve prediction accuracy for small datasets. The CATPCA-SVR model (*R*^2^ = 0.59 and *R* = 0.77) ranked the best among the constructed models. Cha et al. (2022b) compared the performances of several machine learning models for forecasting demolition waste generation in South Korea. The hyperparameters of the developed ANN, RF, KNN, MLR, and SVM models were adjusted to enhance their outcomes. The findings confirmed the outperformance of ANN-ReLu (*R*^2^ = 0.90 and ratio of percent deviation (RPD) = 3.16), SVM polynomial (*R*^2^ = 0.89 and RPD = 3.00), and ANN-logistic (*R*^2^ = 0.88 and RPD = 2.92). The average errors of the developed models were 7.3%, 7.4%, and 7.5%, respectively. Yuan et al. (2023) quantified the urban material stock using data from 71 demolished buildings in Hong Kong. The proposed model accounted for six features: construction year, building type, perimeter, height, total floor area, and number of floors. An MLR model produced a reliable estimate of construction material stockpiles with a root mean square error of 474.13, a mean absolute percentage error of 9.1%, and an *R*^2^ of 0.93 compared to other machine learning models.

On a national level, Elgizawy et al. (2016) compared and evaluated the CDW quantification methods in the literature, highlighting their advantages and shortcomings. The study evaluated the construction waste index for two medium-sized residential projects and two LEED-certified projects in Egypt. The average indices for the former projects were 0.115 ton/m^2^, but the respective indices of the latter projects were roughly 0.025 ton/m^2^ and 0.026 ton/m^2^. The computed index for these small- to medium-sized projects was four times larger than the one for large-scale projects. This index could serve as a foundation for comparing various project types in Egypt and anticipating the quantities of waste generated from future projects. Elshaboury and Attia (2022) modeled the CDW quantities in four Egyptian governorates using six input factors: population size, percentage of residential to nonresidential structures, built-up and demolished areas, and the number of building units. The data was gathered from the Central Agency for Public Mobilization and Statistics for 2010–2019. The developed ANN models yielded an average *R*-value of 0.89, with a typical validity percent value ranging from 0.73 to 0.84. The conclusions demonstrated the capability of ANN models to predict the generated quantities of CDW in El-Gharbia, Kafr El Sheikh, Assiut, and Qena.

The previous research studies have not jointly tackled the following points: (1) investigating the factors influencing the CDW generation in Egypt, (2) quantifying the CDW volume in an Egyptian city using GIS and machine learning, and (3) improving the search capabilities of conventional machine learning models that are constrained by local minima trapping and poor convergence. In this regard, the major objective of this research is to provide a robust methodology for modeling CDW generation rates in Tanta City, one of the largest and most civilized cities in Egypt. The following are the major contributions of this research study: (1) incorporating the socioeconomic and waste-generation statistics to estimate the CDW quantities, (2) employing the classification of satellite images that were acquired over long periods to estimate the urban growth as well as vacant and agricultural areas, (3) forecasting the waste generation using hybrid SOFNN modeling approaches (i.e., AQPSO-SOFNN and SOFNN-HPS), (4) validating the performance of the developed models against state-of-the-art competitors using several evaluation measures, and (5) reducing the mean absolute percentage error (MAPE) and normalized root means square error (NRMSE) metrics of the developed FNN-EBP and GMDH models by at least 92.90% and 90.64%, respectively. This research study can help the government monitor urban metabolism and establish efficient waste management systems in the study region.

## Materials and methods

### Study area

**Fig. 1:**
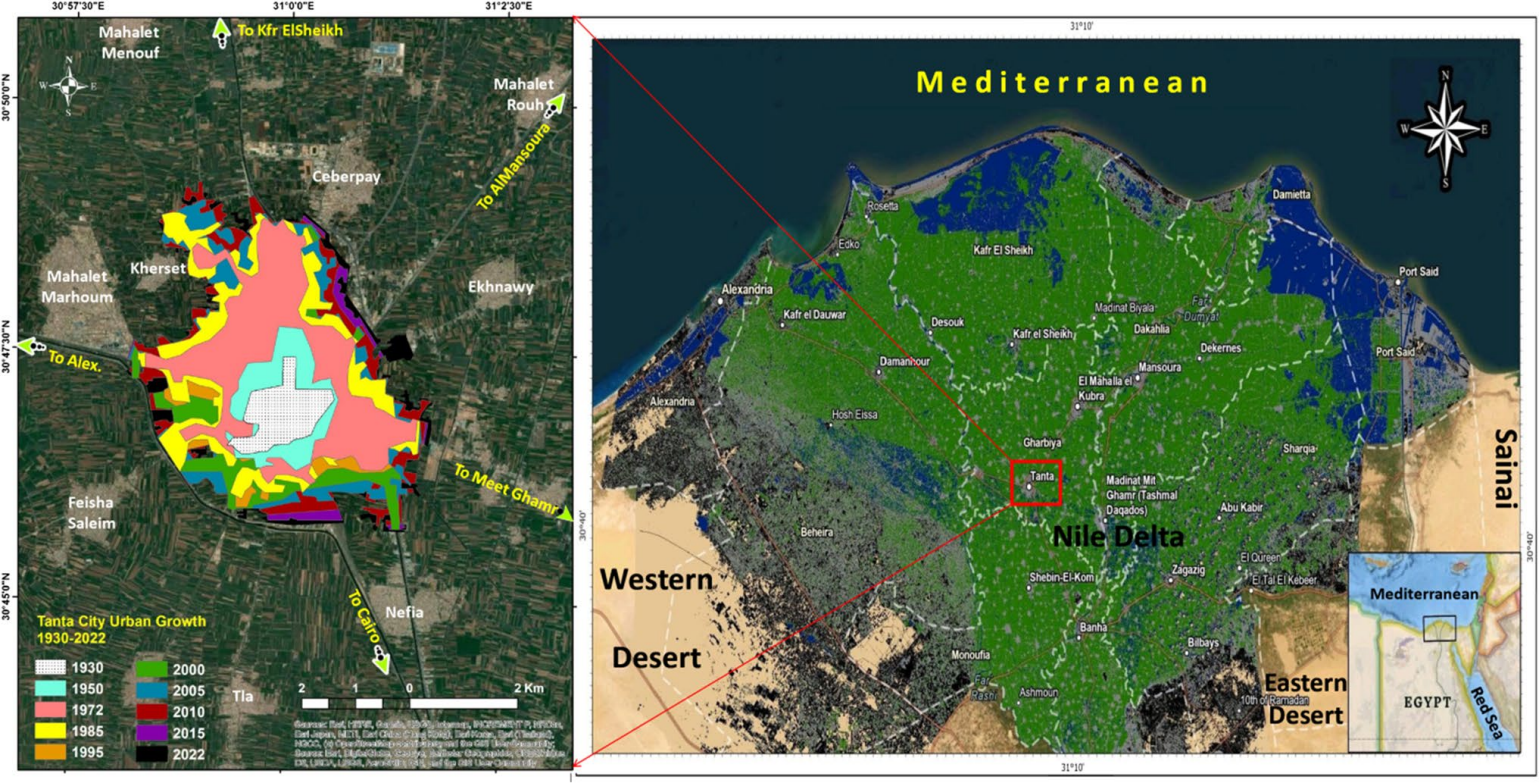
Location and urban growth map of Tanta City in Egypt

Tanta City is the capital of Gharbia governorate, which lies iwest of the Nile Delta and north of Egypt (see Fig. 1). It is located 120 km southeast of Alexandria and 90 km north of Cairo. It is located 10 m above sea level at the latitude 30° 47′ 28″ N and longitude 30° 59′ 53″ E. The city has experienced a fast expansion in urban growth during the past 25 years (Abdrabo et al. 2021). It has the largest area and population size in the Delta, in addition to being the most civilized city after Cairo, the capital, and Alexandria. Between 1996 and 2017, the population nearly expanded 1.4 times, and it is anticipated to reach 558,383 people in 2027. On a global level, it came in second place in the list of the most densely populated cities in the world with 27,800 inhabitants/km^2^ for the year 2022 (Egypt Independent 2022). This figure can be attributed to the nonexistence of a desert hinterland and the fact that the city is surrounded by an agricultural hinterland, limiting its expansion opportunities. The problem is compounded by the urban sprawl on agricultural lands and construction violations following the 25 January revolution in 2011, exacerbating congestion in the city (Mostafa et al. 2023). In this regard, the city can either amend the administrative borders of the governorate, which are considered only suitable in the medium and long term, or utilize the vacant areas such as illegal waste disposal areas. The second option is more applicable shortly, as it allows for establishing services that offer people a high quality of life and reduce the adverse effects of overpopulation (Elwatan News 2022).

### Research methodology

**Fig. 2:**
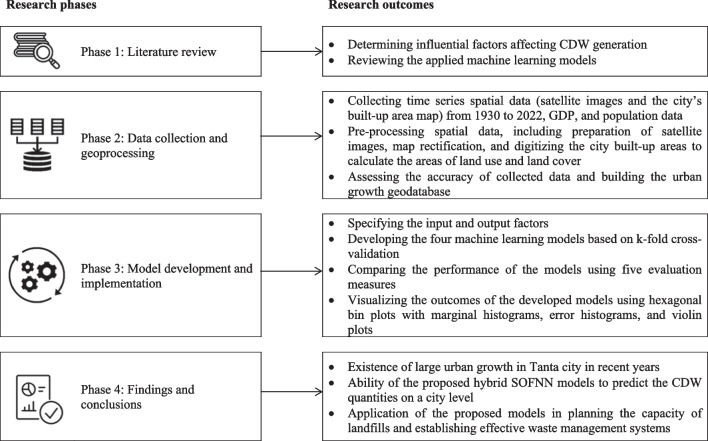
Flowchart of the research study

**Fig. 3:**
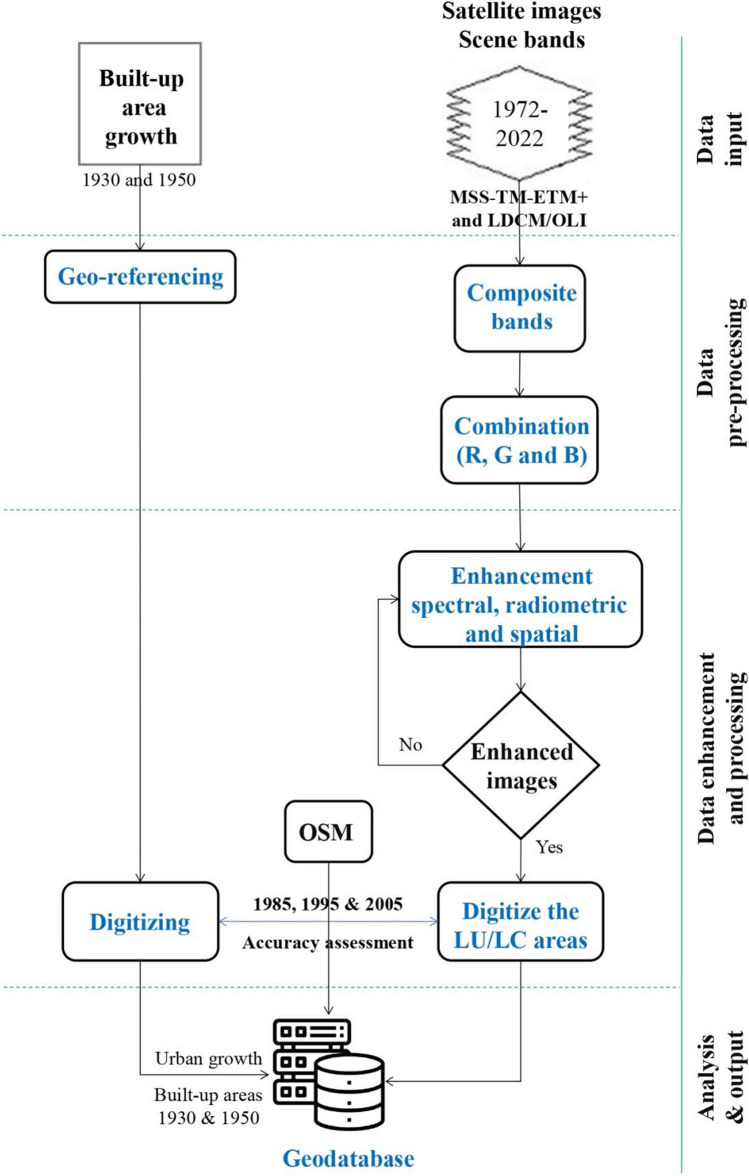
Geo-processing and analysis of the built-up, vacant, and agricultural areas

The proposed flowchart for estimating the waste quantities starts by defining the influential factors such as year, population, gross domestic product (GDP) per capita, built-up area, vacant area, and agricultural area. Figure 2 presents the research methodology phases and the reported outcomes. The types, formats, and sources of the utilized data are depicted in Table 1 and described as follows: Time series of the city population (capita) and the GDP per capita ($/capita) for the period 1965–2021 are obtained from the city population (2023) and the World Bank (2023), respectively. The detailed geoprocessing and analysis of the remaining factors are presented in Fig. 3. The built-up areas (m^2^) for the period 1930–1950 are acquired based on El-Kholei et al.’s (2016) map that is rectified (according to UTM zone 36 N projection, WGS 1984 datum) and digitized as a polygon feature class geographical database. The areas from 1975 to 2022 are acquired based on the geoprocessing and analysis of satellite images. Table 2 describes the spectral characteristics and spatial resolution of the utilized satellite images.

**Table 1:** Types, formats, and sources of the utilized data

Data	Type	Format	Source
Statistics (population, GDP, and CDW quantities)	Table	.xlsx	City Population (2023), World Bank (2023), and WMRA (2017)
City built-up areas (1985, 1995, and 2005)	Raster	Paper (scanned and converted to digital jpg format)	El-Kholei et al. (2016)
Satellite images (1972–2022)	Raster	Geo-tiff format	https://earthexplorer.usgs.gov/
Topographic map (2 sheets), No. NH36-16c and NH36-15d, scale 50,000	Raster	Paper (scanned and converted to digital jpg format)	Egyptian General Survey Authority, Tanta east and west map sheets (1997), 50,000, Ministry of Water Resources and Irrigation, Cairo, Egypt
Study area/administrative unit boundary	Vector	Shapefile	https://www.diva-gis.org/Data based on https://gadm.org/ (version 1.0)
Base map	OSM	Map tiles	https://www.openstreetmap.org/

**Table 2:** Spectral characteristics and spatial resolution of the utilized satellite images

Satellite	Sensor and spectral resolution (µM)	Band	Spatial resolution (M.)	Swath (km)	Scene size (km × km)	Altitude (km)
Landsat 5	TM					
	Band 1: 0.45–0.52	Blue	30	185	170 × 183	705
	Band 2: 0.52–0.60	Green	30			
	Band 3: 0.63–0.69	Red	30			
	Band 4: 0.76–0.90	Near IR	30			
	Band 5: 1.55–1.75	Mid IR	30			
	Band 6: 10.4–12.5	Thermal	120			
	Band 7: 2.08–2.35	Mid IR	30			
Landsat 7	ETM +					
	Band 1: 0.450–0.515	Blue	30	185	170 × 183	705
	Band 2: 0.525–0.605	Green	30			
	Band 3: 0.630–0.690	Red	30			
	Band 4: 0.760–0.900	Near IR	30			
	Band 5: 1.550–1.750	Mid IR	30			
	Band 6^a^: 10.40–12.5	Thermal	60			
	Band 7: 2.080–2.35	Mid IR	30			
	Band 8: 0.52–0.92	Pan	15			
Landsat 8 and 9	OLI					
	Band 1: 0.43–0.45	Visible	30	185	170 × 185	705
	Band 2: 0.450–0.51	Visible	30			
	Band 3: 0.53–0.59	Visible	30			
	Band 4: 0.64–0.67	Red	30			
	Band 5: 0.85–0.88	Near-IR	30			
	Band 6: 1.57–1.65	SWIR 1	30			
	Band 7: 2.11–2.29	SWIR 2	30			
	Band 8: 0.50–0.68	PAN	15			
	Band 9: 1.36–1.38	Cirrus	30			
	TIRS					
	Band 10: 10.6–11.19	TIRS 1	100			
	Band 11: 11.5–12.51	TIRS 2	100			

^a^Band 6 on Landsat 7 is divided into 2 bands, high and low gain

Source: (http://glcf.umd.edu/) [scene pass 177/row 039]

The accuracy assessment process of satellite image classification is applied using topographic maps and OpenStreetMap (OSM) in addition to El-Kholei et al.’s (2016) map to validate the years 1985, 1995, and 2005. As such, the built-up area for the study period is calculated based on the urban growth formula shown in Eq. (1), assuming the stability of the rest of the factors affecting urban growth.

1 $$R=\left[\left({V}_{\mathrm{present}}-{V}_{\mathrm{past}}\right)/{V}_{\mathrm{past}}\right]\times 100$$

where $R$, ${V}_{\mathrm{present}}$, and ${V}_{\mathrm{past}}$ denote the growth rate, present or future value, and past or present value, respectively. It shall be noted that the annual growth rate is simply the percent growth divided by the number of years, considering that the computed rate represents the average of rates over different periods.

The vacant and agricultural areas (m^2^) are deduced from several land use/land cover maps that are extracted from the visual interpretation and classification of satellite images. The percentage of vacant areas is determined by studying the map of land uses in different periods. Moreover, there is a decrease in agricultural areas resulting from the growth of the built-up area over the study period. As such, the vacant and agricultural areas are deduced for the subsequent periods under investigation through area field calculations within the attribute tables of multi-temporal land use/land cover layers in the geographical database. These attribute tables are generated automatically when the geographical database layer type is specified as a feature class (polygon) and structured under a metric coordinate framework (here, UTM-WGS 1984 zone 36 N). The waste quantities are extracted from the report published by the waste management regulatory authority for the Gharbia governorate (WMRA 2017).

After specifying the input and output factors, the data are analyzed using the AQPSO-SOFNN, SOFNN-HPS, FNN-EBP, and GMDH models. These models are employed to forecast the waste generation rates in Tanta City based on k-fold cross-validation. Cross-validation assesses how effectively the proposed machine learning models can forecast the outcome of new data. The AQPSO-SOFNN employs a cooperative adaptive adjustment approach for attractor, coefficient, and boundary to achieve the optimal balance between exploration and exploitation of the algorithm. It uses an improved fuzzy recursive least squares technique to determine the nonlinear dynamical system. Finally, the Lyapunov stability theory is used to demonstrate the convergence of the proposed model precisely. The SOFNN-HPS algorithm devises an online self-organizing strategy for concurrently determining the network topology and parameters to achieve the optimum balance between system accuracy and network complexity. Moreover, it utilizes an adaptive allocation strategy to decide the antecedent parameters of fuzzy rules. The resultant parameters of the derived fuzzy rules are then updated using a modified recursive least squares technique to hasten the convergence of the error. Finally, after initializing the model parameters, the models are developed and assessed using five metrics to identify the optimum prediction model.

### Machine learning models

Four machine learning models are applied to predict the CDW quantities in Tanta City, Egypt. Each of these models is described in detail in the next subsections.

#### AQPSO-SOFNN

In this study, the SOFNN architecture has four layers: the input layer, the membership function layer, the rule layer, and the output layer (Zhang and Wang 2022). The input layer comprises neurons, each representing a different input variable. This layer requires no weight modifications because the input neuron is transmitted directly to the next layer. Each neuron in the membership function layer acts as a membership function to carry out the fuzzification method. Each neuron in the rule layer corresponds to a fuzzy rule’s IF part (antecedent parameters). The output variable is the weighted summation of input signals, and it is generated using a defuzzification algorithm.

**Fig. 4:**
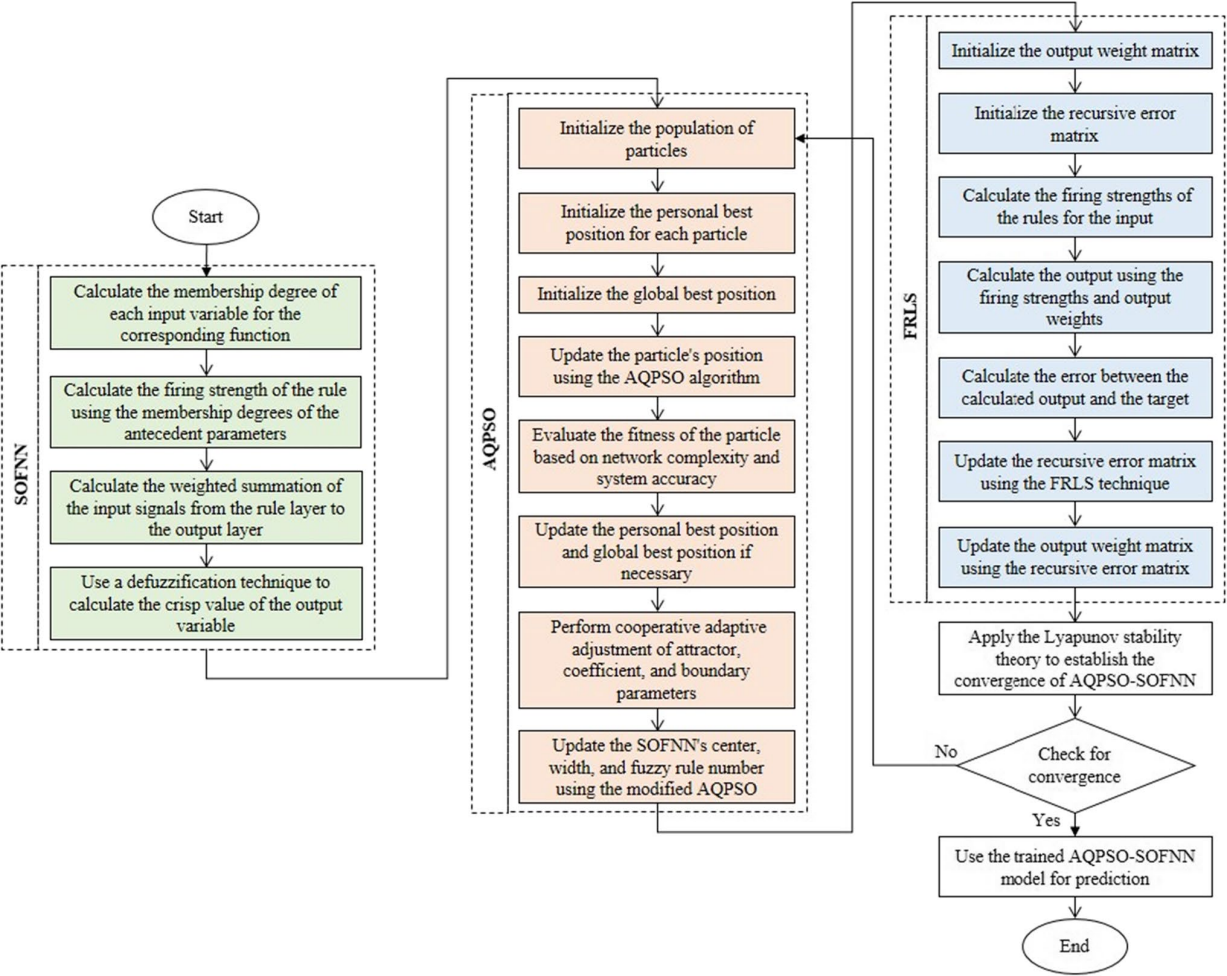
Flowchart of AQPSO-SOFNN model

The basic background and procedures of this algorithm are described as illustrated in Fig. 4 (Zhou et al. 2022). Compared to particle swarm optimization (PSO), quantum PSO (QPSO) eliminates particle velocity information and delivers better search performance with a more streamlined model structure. However, QPSO also encounters premature convergence and entrapment in a local optimum. To this end, a cooperative adaptive adjustment technique for the attractor, the coefficient, and the boundary is proposed. The resulting AQPSO algorithm balances exploration and exploitation capacities. The global optimal solution is found using this approach, enhancing the precision of the solution. The free parameters and network topology are updated simultaneously by building the fitness function, utilizing the network complexity and system accuracy during the learning process. An improved fuzzy recursive least squares (FRLS) technique is subsequently presented to estimate the output weights of SOFNN. Also, the Lyapunov stability theory establishes the convergence of the AQPSO-SOFNN to guarantee its ability to solve real-world engineering problems. The center, width, and fuzzy rule number of the SOFNN are finally modified using the suggested AQPSO.

#### SOFNN-HPS

**Fig. 5:**
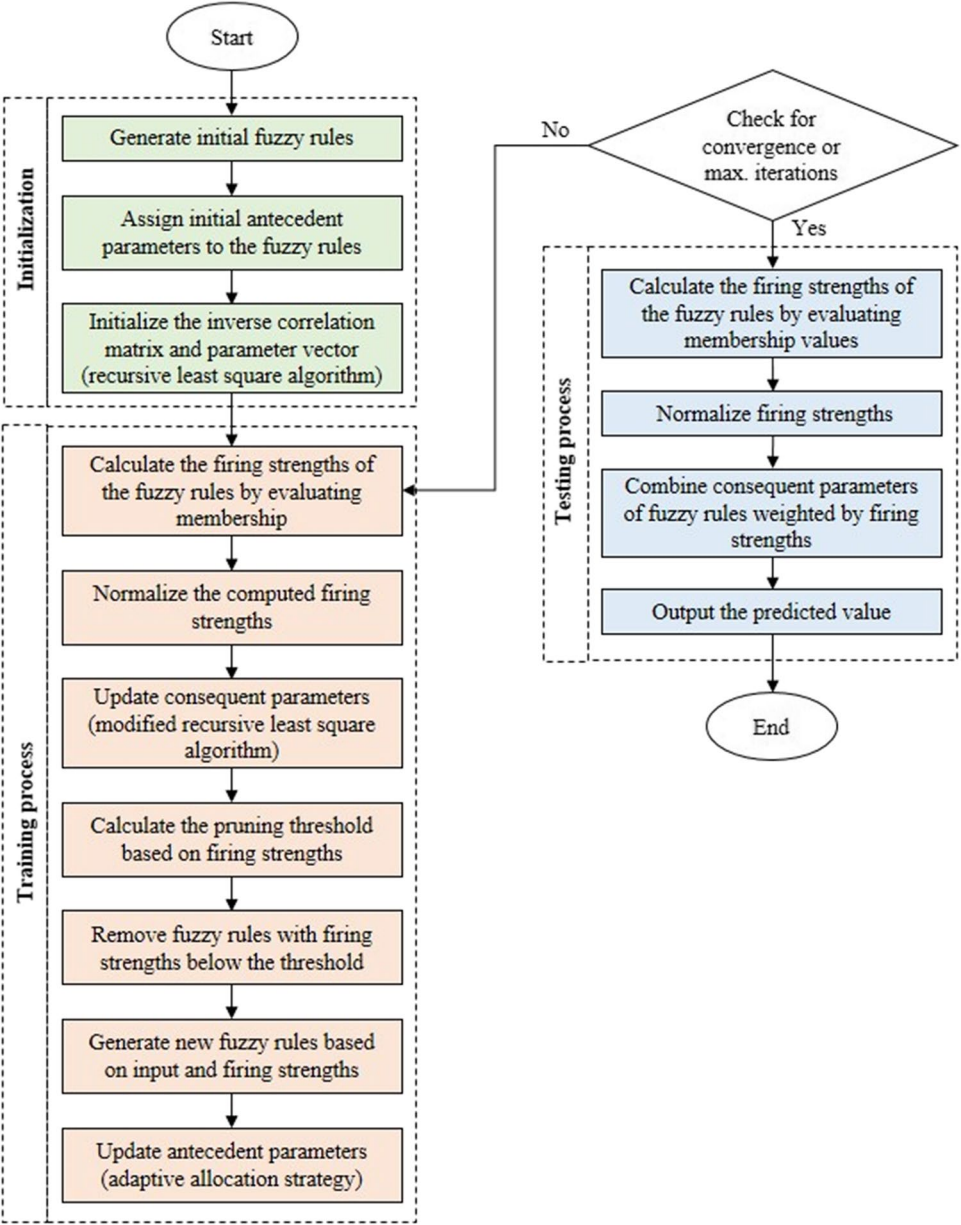
Flowchart of SOFNN-HPS model

SOFNN based on a HPS is proposed to attain the ideal balance between network accuracy and complexity by adjusting the network topology and parameters (Zhou et al. 2020). The procedures of SOFNN-HPS are described in Fig. 5 as follows: The capacity of fuzzy rules to characterize nonlinear systems is enhanced using asymmetric Gaussian functions that can split the input space. HPS is used to create fuzzy rules while automatically eliminating redundant fuzzy rules without pre-setting the pruning threshold or inadvertently deleting significant rules. Finally, the fuzzy rules’ antecedent parameters are chosen using an adaptive allocation technique throughout the learning process. By modifying the area of the generalized ellipsoidal basis functions, this method strikes a compromise between rule-base interpretability and accuracy for a better local approximation. The resultant parameters of the obtained fuzzy rules are finally updated using a modified recursive least squares method to hasten the convergence of the estimation error.

#### FNN-EBP

The FNN-EBP learning approach comprises fuzzy processing and a standard BP network. The membership function in the fuzzy processing component applies fuzzy processing to the network input, and the results are sent to the BP network for additional processing. The output data are compared to the expected output to determine the network connection weights, which are then modified based on an error function (Li et al. 2011). The FNN architecture comprises the input layer, the membership function layer, the rule layer, and the output layer. The neurons in the input layer are sent directly to the following layer without any weight modifications. Meanwhile, neurons in the membership function layer are responsible for the fuzzification process. The symmetric width constraint of the Gaussian function is circumvented by using an asymmetric Gaussian function with dynamic widths. This layer has the same number of neurons and fuzzy rules. A defuzzification method determines the output variable, which is the weighted sum of the input signals. The FNN-EBP network structure expands when additional fuzzy rules are specified without improving the algorithm’s performance. On the other hand, applying fewer fuzzy rules might compromise the generalization performance and prediction accuracy of the model.

#### GMDH

A self-organized system called the GMDH was introduced for dealing with nonlinear problems (Ivakhnenko 1971). It identifies data relationships, selects the ideal structure or network design, and enhances the precision of existing techniques while considering all conceivable input combinations. A quadratic polynomial connects the neurons in each layer, generating new neurons in the subsequent layers. Even though standard GMDH offers systematic prediction and system modeling, there are still several limitations to its use (Mulashani et al. 2022): (1) difficulty in determining the best dataset split and elimination of important features resulting in slight differences in the model accuracy, (2) high propensity to produce excessively intricate solutions when handling nonlinear problems due to its generic architecture (quadratic polynomial), (3) difficulty of determining neuron weights by a quadratic polynomial, (4) unfit convergence or divergence caused by the incorrect selection of the variables, and (5) the need to conduct several runs using different initial assumptions to obtain the global optimum.

### Performance metrics

In this research, MAPE, NRMSE, *R*^2^, Kling–Gupta efficiency (KGE), and index of agreement (IOA) metrics are utilized to assess the performance of the proposed models as per Eqs. (2)–(6). The MAPE measures the magnitude of errors (in percentage terms) encountered by the developed models in predicting the outcomes. The NRMSE is the proportion of the RMSE related to the range of the observed value. Lower values of MAPE and NRMSE indicate more accurate predictions from the developed model, and vice versa. The *R*^2^ value measures the performance of the prediction model to predict an outcome in linear regression. The KGE metric is a revised version of the Nash–Sutcliffe efficiency to prevent the association between bias and variability ratios. Finally, the ratio between the mean square error and the potential error is represented by the IOA. It can detect changes between the observed and predicted values, but it is sensitive to extreme values because of the squared differences. Higher values of *R*^2^, KGE, and IOA metrics indicate robust model performance and vice versa.

2 $$\mathrm{MAPE}=100\times \frac{1}{n}\times \sum_{i=1}^{n}\left|\frac{{O}_{i}-{P}_{i}}{{O}_{i}}\right|$$

3 $$\mathrm{NRMSE}=\frac{\mathrm{RMSE}}{{O}_{i}}$$

4 $${R}^{2}={\left[\frac{\sum_{i=1}^{n}\left({O}_{i}-\overline{{O }_{i}}\right)\left({P}_{i}-\overline{{P }_{i}}\right)}{\sqrt{\sum_{i=1}^{n}{\left({O}_{i}-\overline{{O }_{i}}\right)}^{2}} \sqrt{\sum_{i=1}^{n}{\left({P}_{i}-\overline{{P }_{i}}\right)}^{2}}}\right]}^{2}$$

5 $$\mathrm{KGE}=1-\sqrt{{\left(R-1\right)}^{2}+{\left(\beta -1\right)}^{2}+{\left(\gamma -1\right)}^{2}}, \beta =\overline{{P }_{i}}/\overline{{O }_{i}}, \gamma =\left({\sigma }_{p}/\overline{{P }_{i}}\right)/\left({\sigma }_{o}/\overline{{O }_{i}}\right)$$

6 $$\mathrm{IOA}=1-\frac{\sum_{i=1}^{n}{\left({O}_{i}-{P}_{i}\right)}^{2}}{\sum_{i=1}^{n}{\left(\left|{P}_{i}-\overline{{O }_{i}}\right|+\left|{O}_{i}-\overline{{O }_{i}}\right|\right)}^{2}}$$

where $n$ is the number of available data records, ${O}_{i}$ is the observed waste quantities in the *i*th year, and ${P}_{i}$ is the predicted waste quantities using the developed models in the *i*th year.

## Results and discussion

The main input factors include the year, population, GDP per capita, built-up area, vacant area, and agriculture area, while the output factor denotes the quantities of waste. The input and output sample data are handled using four machine learning models to accomplish the nonlinear approximation of the input factors to the CDW quantities. A total of 57 data records were gathered in Tanta City, Egypt. A fivefold cross-validation was conducted to validate the outcomes of the developed models. The hyperparameters of the AQPSO-SOFNN are outlined as follows: balance factor = 0.4, population size = 50, and maximum number of steps = 200. Considering the SOFNN-HPS, it is assumed that the maximum error = 1.0, the pruning cycle = 50, the desired precision = 0.09, and the minimum and maximum lengths of the input space = 0.1 and 1.0, respectively. The control parameters of the FNN-EBP are defined such that the rule number = 10 and the learning rate = 0.001. For the GMDH, the maximum number of layers = 2, the maximum number of neurons in a layer = 10, and the selection pressure (in layers) = 0.6. Additionally, the maximum number of iterations is set at 100 to ensure a fair comparison between the performances of the models. MATLAB version R2019a is utilized to perform the proposed models.

**Fig. 6:**
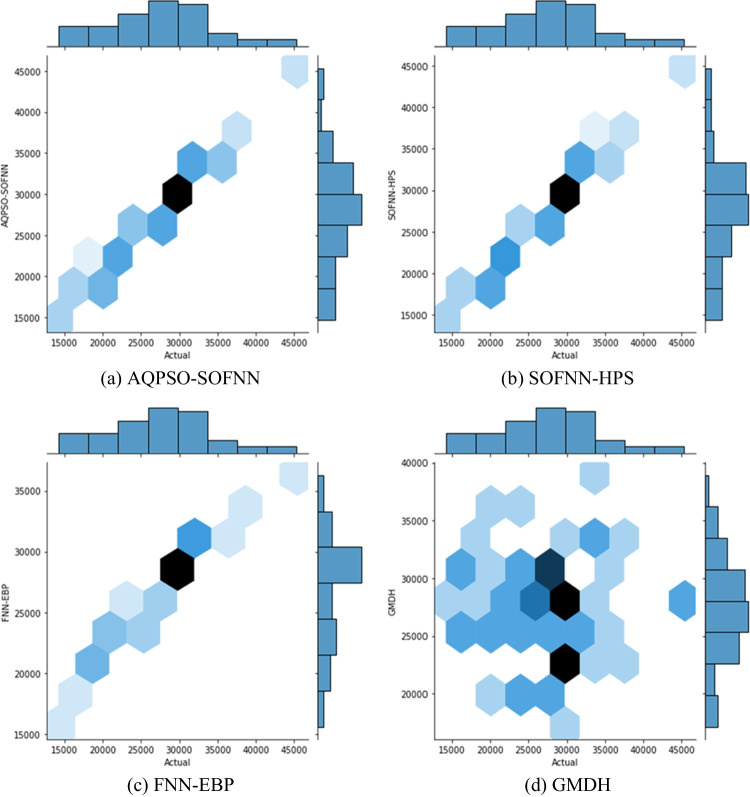
Hexagonal bin plots with marginal histograms of the observed and predicted waste quantities using the machine learning models

The performance of the developed models is assessed using hexagonal bin plots with marginal histograms (Fig. 6). Each hexagonal bin in these plots represents a cluster of data points, with the *x*-axis indicating observed quantities and the *y*-axis representing predicted quantities. Meanwhile, the color intensity shows the density of data points, allowing the detection of high-concentration areas and prediction accuracy patterns.

By comparing the predictions of four models, it can be shown that AQPSO-SOFNN predicts waste quantities with a substantially higher degree of accuracy than FNN-EBP and GMDH. The AQPSO-SOFNN hexagonal bins are more densely concentrated around the 45° regression line, suggesting a closer match between observed and predicted quantities. Despite the outperformance of this model, the SOFNN-HPS model also demonstrates comparable performance, with hexagonal bins closely aligned with the diagonal line. The AQPSO-SOFNN and SOFNN-HPS models have close equivalence to the observed values for the entire dataset. As a result, these models demonstrate high performance in estimating waste volumes compared to other models.

The comparison goes beyond hexagonal bins, and the marginal histograms reflect the distribution of errors along the *x*-axis and *y*-axis. This allows for analyzing the overall distribution of errors and identifying biases in predictions.

The fivefold cross-validation average predictive performances of the four machine learning models are compared using MAPE, NRMSE, *R*^2^, KGE, and IOA evaluation metrics, as depicted in Table 3. The AQPSO-SOFNN model is associated with a maximum determination coefficient of 1.00. This implies a perfect alignment between observed and predicted values, demonstrating the model’s outstanding predictive capacity. Meanwhile, the KGE and IOA metrics are equal to 0.99 and 1.00 for the AQPSO-SOFNN and SOFNN-HPS models, respectively. As for the FNN-EBP and GMDH models, the *R*^2^, KGE, and IOA values lie in the range of [0.02, 0.95], [0.01, 0.67], and [− 0.03, 0.95], respectively. The varying performance values represent the different degrees of accuracy across the models. The same results can be interpreted by checking the average MAPE and NRMSE of the four comparative algorithms. The proposed AQPSO-SOFNN model reduces the MAPE by 55.13%, 92.90%, and 98.43% compared to the SOFNN-HPS, FNN-EBP, and GMDH, respectively. Additionally, it reduces NRMSE by more than 55.13% compared to all the other models. The simulation outcomes show that the AQPSO-SOFNN model can accurately predict the CDW quantities.

**Table 3:** Average performance of the developed machine learning models for the entire dataset

Metrics	AQPSO-SOFNN	SOFNN-HPS	FNN-EBP	GMDH
First fold				
MAPE	1.50%	3.50%	11.40%	47.70%
NRMSE	0.02	0.06	0.134	0.511
*R*^2^	1.00	0.98	0.98	0.01
KGE	0.99	0.94	0.64	− 0.07
IOA	1.00	0.99	0.95	− 0.16
Second fold				
MAPE	0.70%	0.00%	10.10%	49.50%
NRMSE	0.01	0.00	0.13	0.53
*R*^2^	1.00	1.00	0.98	0.02
KGE	1.00	1.00	0.66	− 0.14
IOA	1.00	1.00	0.95	− 0.31
Third fold				
MAPE	0.60%	0.00%	7.70%	39.70%
NRMSE	0.01	0.00	0.10	0.45
*R*^2^	1.00	1.00	0.99	0.03
KGE	0.99	1.00	0.746	0.17
IOA	1.00	1.00	0.98	0.29
Fourth fold				
MAPE	0.70%	0.00%	8.80%	50.10%
NRMSE	0.02	0.00	0.13	0.53
*R*^2^	1.00	1.00	0.93	0.03
KGE	0.99	1.00	0.70	− 0.17
IOA	1.00	1.00	0.95	− 0.40
Fifth fold				
MAPE	4.30%	0.00%	11.30%	35.70%
NRMSE	0.07	0.00	0.16	0.42
*R*^2^	0.96	1.00	0.87	0.074
KGE	0.97	1.00	0.66	0.27
IOA	0.99	1.00	0.92	0.43
Average				
MAPE	0.70%	1.56%	9.86%	44.54%
NRMSE	0.01	0.03	0.13	0.49
*R*^2^	1.00	0.99	0.95	0.02
KGE	0.99	0.99	0.67	0.01
IOA	1.00	1.00	0.95	− 0.03

**Fig. 7:**
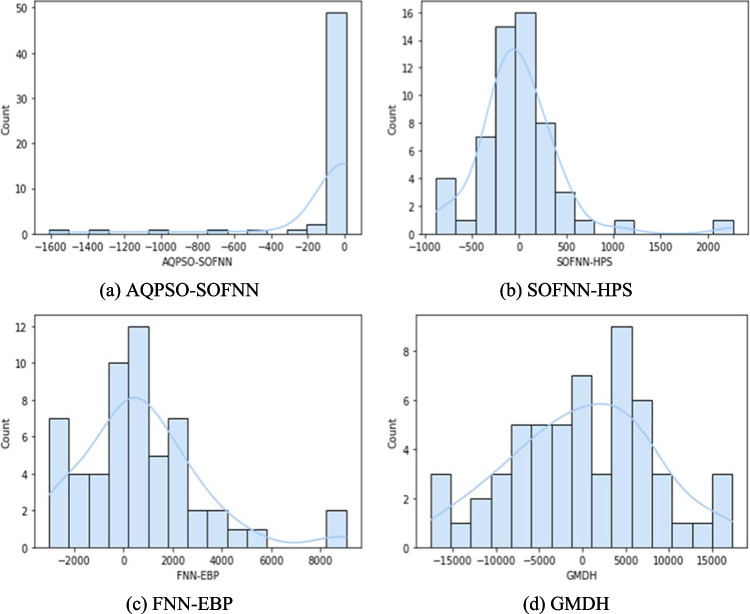
Error histograms of waste quantities using the developed models for the entire dataset

The error histogram diagrams for the complete dataset are shown in Fig. 7. As depicted in the figure, the *x*-axis reflects the error values, while the *y*-axis shows the frequency of each error value. The frequency and distribution of errors within the histogram give useful information about the performance of prediction models. The frequency of errors approaches zero as modeling accuracy improves. In light of this, the optimal modeling performance is represented by the bell-shaped error histogram with a mean of zero. This signifies that the predictions closely match the observed values. It is obvious that the AQPSO-SOFNN model has the least errors, as evidenced by its tighter distribution around zero error. Following this, the SOFNN-HPS, FNN-EBP, and GMDH models exhibit broader distributions, indicating disparities between predictions and observations. However, the FNN-EBP and GMDH models have a higher frequency of nonzero errors.

Moreover, the histogram reveals the nature of errors, discriminating between overestimation and underestimation. Overestimation and underestimation are indicated by positive and negative errors, respectively. The AQPSO-SOFNN and SOFNN-HPS models underestimate the waste quantities, unlike the FNN-EBP, which mostly overestimates the outputs. Additionally, the GMDH model shows both underestimation and overestimation of the outcomes.

Additionally, the histogram provides insights into the magnitude of errors by visualizing the range of errors for each model on the y-axis. The errors of the AQPSO-SOFNN, SOFNN-HPS, FNN-EBP, and GMDH range between [− 0.0001, 9.13], [− 17.22, 2267.23], [− 65.79, 9048.78], and [− 74.59, 17,343.53], respectively. These ranges emphasize the capacity of the AQPSO-SOFNN model to minimize errors.

**Fig. 8:**
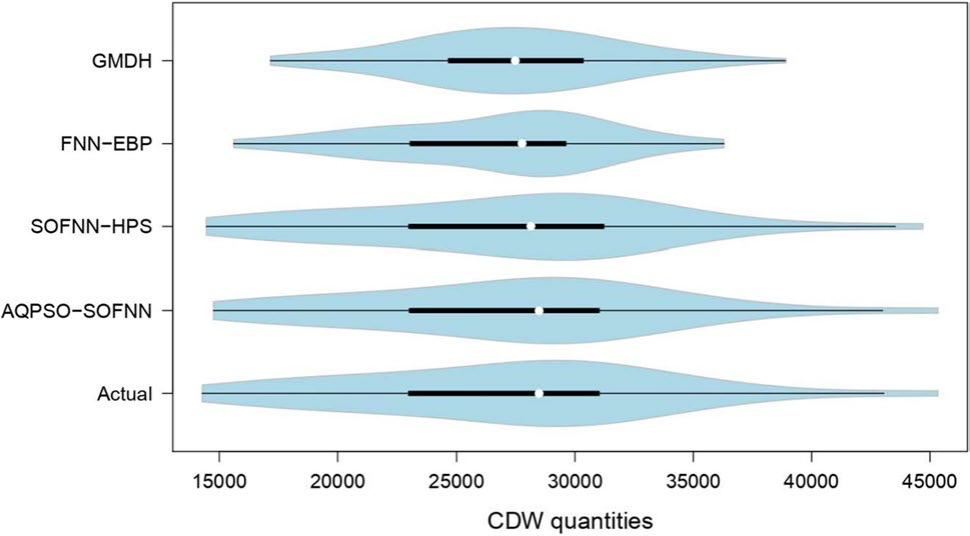
Violin plots for comparing observed and modeled waste quantities using machine learning models

The violin plot diagram of the observed and forecasted waste quantities is displayed in Fig. 8. Examining the resemblance between the distribution of observed and modeled waste quantities is the primary function of this plot. The modeling accuracy increases in case the modeled distribution resembles the shape of the observed waste quantities. The distribution of the AQPSO-SOFNN and SOFNN-HPS models closely fits that of the observed data, given that the medians of the modeled and observed data point in the same direction. The same lengths of interquartile ranges show similar data dispersion for both models. The FNN-EBP comes in third place, followed by the GMDH, whose distribution differs significantly from the observed data. The AQPSO-SOFNN and SOFNN-HPS models rely on fuzzy neural networks, and as such, they have a greater capacity to identify nonlinear systems than other networks. The former model employs a cooperative adaptive adjustment approach, an improved FRLS technique, and Lyapunov stability theory to enhance its nonlinear dynamical approximation capability. Furthermore, the latter model has strong ability and robustness as a result of integrating the hierarchical pruning strategy, the adaptive allocation strategy, the generalized ellipsoidal basis function, and the modified recursive least squares method.

The results of the present study are compared to those reported in the literature. The AQPSO-SOFNN and SOFNN-HPS models have an average *R*^2^ of 0.995 and offer more precise predictions than the deep learning models developed by Akanbi et al. (2020), which had a *R*^2^ value of 0.97 and MAE ranging between 17.93 and 19.04. The same interpretations can be found by investigating Cha et al.’s (2020) study that employed the random forest model (*R* = 0.69–0.87 and *R*^2^ = 0.55–0.80) to predict demolition waste generation. Additionally, the proposed models outperformed the CATPCA-SVR model (*R*^2^ = 0.59, *R* = 0.77) developed by Cha et al. (2022a) and the ANN-ReLu (*R*^2^ = 0.90 and RPD = 3.16), SVM-polynomial (*R*^2^ = 0.89 and RPD = 3.00), and ANN-logistic (*R*^2^ = 0.88 and RPD = 2.9) models that were employed by Cha et al. (2022b) to predict the generation rates of demolition waste.

## Conclusion

The building and construction industry is crucial to the economic development of any country. It consumes resources and raw materials, pollutes the environment, and generates waste. To ensure effective CDW management, governments and local authorities must have precise estimates of waste generation rates because waste quantification is necessary for many waste management strategies, including designing landfill space, enacting disposal levies or recycling subsidies, and developing waste management plans. In this regard, waste quantification prediction models may be developed utilizing socioeconomic, urban growth, and waste-generation statistics. This research applies the AQPSO-SOFNN and SOFNN-HPS models to model the generated CDW quantities in Tanta City, Gharbia governorate, Egypt. The outperformance of the proposed models is exhibited by comparing their performances against those of the FNN-EBP and GMDH models using five evaluation measures: MAPE, NRMSE, *R*^2^, KGE, and IOA. For the AQPSO-SOFNN and SOFNN-HPS models, the KGE and IOA metrics are equivalent to 0.99 and 1.00, respectively. The *R*^2^, KGE, and IOA values for the GMDH and FNN-EBP models lie in the range of [0.02, 0.95], [0.01, 0.67], and [− 0.03, 0.95], respectively. The average MAPE and NRMSE of the four prediction models can be used to interpret the same results. The suggested AQPSO-SOFNN model decreases the NRMSE by at least 55.13% in addition to reducing the MAPE for the SOFNN-HPS, FNN-EBP, and GMDH by 55.13%, 92.90%, and 98.43%, respectively, when compared to other models. The simulation results demonstrated that the AQPSO-SOFNN model performs much better when compared to other comparative models and slightly better than the SOFNN-HPS model. This paves the way for the model’s application in predicting waste quantities at the city level. This can be attributed to the higher capacity of AQPSO-SOFNN and SOFNN-HPS to identify nonlinear systems than other networks. The former model increases its capacity for nonlinear dynamical approximation by incorporating a cooperative adaptive adjustment strategy, a modified FRLS method, and Lyapunov stability theory. The latter model, however, combines the HPS, the adaptive allocation strategy, the generalized ellipsoidal basis function, and the modified recursive least squares technique, offering considerable ability and robustness. Owing to the enormous volumes of generated waste and the scarcity of landfills, it is acknowledged that the proposed methodology for estimating the CDW quantities at a city level can assist the government in planning the optimal capacity of required landfills and in establishing the necessary legislation for effective waste management systems. However, certain limitations in the applicability of our methodology to other contexts must be acknowledged. The current methodology’s performance is optimized for Tanta City and may require modification when applied to other cities with different characteristics or data availability. Factors including building location, type (e.g., residential and commercial), and structure (e.g., reinforced concrete, brick, and wood) can all impact the model’s performance. Future studies might focus on conducting comparative studies across different cities, regions, or countries to examine differences in CDW generation rates, waste management strategies, and policy implications. This could offer valuable insights into the factors driving waste management efficacy.

## Data Availability

The datasets used and/or analyzed during the current study are available from the corresponding author upon reasonable request.
